# Unified promoter opening steps in eukaryotic gene expression

**DOI:** 10.18632/oncotarget.21387

**Published:** 2017-09-29

**Authors:** Jérémy Sandoz, Frédéric Coin

**Affiliations:** Frédéric Coin: IGBMC, Department of Functional Genomics and Cancer, CNRS/INSERM/University of Strasbourg, Illkirch, C.U. Strasbourg, France; Centre National de la Recherche Scientifique, UMR7104, Illkirch, France; Institut National de la Santé et de la Recherche Médicale, U964, Illkirch, France; Université de Strasbourg, Illkirch, France

**Keywords:** TFIIH, XPB, transcription, promoter opening

**Figure 1:**
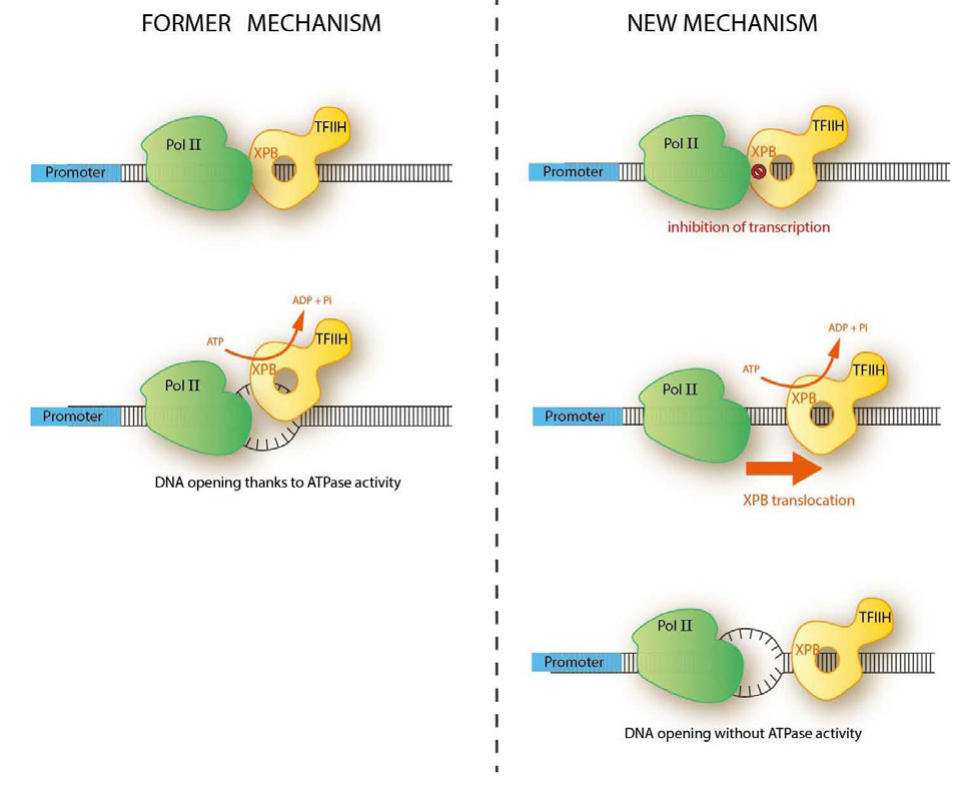
Former and new mechanisms of promoter opening in Pol II-dependent gene expression.

The interest of scientists for the transcription of the eukaryotic genome started four decades ago with the purification of the three RNA polymerase (Pol) enzymes. Pol I, II and III transcribe different species of RNA with the help of several general/basal transcription factors (TFs). Messenger RNA (mRNA) transcription by Pol II has been intensely studied owing first to the protein-coding function of mRNA and also to the apparent complexity of this system compared to the transcription of ribosomal RNAs by Pol I or small RNAs by Pol III. Indeed, it was shown very early that mRNA transcription required, apart from Pol II, additional enzymatic activities that didn’t seem to be needed in the Pol I or III systems. Surprisingly enough, all these activities reside in TFIIH, a multi-subunit transcription/repair factor that is involved, through its CDK7 and XPB subunits, both in the phosphorylation of the carboxyl-terminal domain of the largest Pol II subunit and in the ATP-dependent opening of the promoter around the transcription start site, respectively [3]. Consequently, the model of promoter opening that has prevailed in class II gene expression during the last four decades suggests that XPB was an ATP-dependent DNA helicase that unwinds promoter DNA from -8 to +2, relative to the transcription start site (Figure 1, former model). Since transcription initiation takes place in the absence of ATP in Pol I- and III dependent transcription, it was expected that these enzymes used different molecular mechanisms to open their promoters, using the energy generated during the formation of their PIC.

Recent results contradicted the above Pol II model and have deeply modified our view of transcription initiation in eukaryotes. First of all, in contrast to its ATPase activity that is robust, the *in vitro* helicase activity of XPB appeared very low [3]. Rather, XPB showed the structural and biochemical characteristics of a translocase, able to move along the double stranded DNA without unwinding it [3].

Moreover, technical advances in our capacity to localize transcription factors inside the preinitiation complex (PIC) have revealed that XPB was binding to the promoter DNA downstream from the transcription start site, a location that was not compatible with its role in promoter opening [3]. In addition, recent structural studies revealed that the Pol I and III machineries contained a core that was structurally and functionally conserved in the Pol II initiation complex, pinpointing to a possible convergence of the three systems to a single mechanism of transcription initiation [8]. The final blow to the canonical model of helicase-dependent mechanism of class II promoter opening came from the discovery that Pol II-dependent transcription was sensitive to the inhibition of XPB ATPase activity but accommodated to the absence of the protein [1]. These observations, seemingly contradictory, become meaningful when one takes into consideration the current knowledge about transcription initiation described above. Indeed, they all converge to a model in which XPB initially acts as an inhibitor of promoter opening, blocking the ATP-independent unwinding of the promoter generated by the formation of the PIC. In a second step, XPB uses ATP to translocate along the double stranded DNA downstream from the transcription start site. This movement liberates the promoter and allows its helicase-free Pol I/III-like opening (Figure 1). This model implies that Pol I, II and III do not differ mechanistically from each other on the way promoters are opened but rather on the presence of a regulatory step in Pol II-dependent transcription that controls in time and space promoter opening after the formation of the PIC.

The new regulatory step described above depends on the ATPase activity of XPB and is probably the target of the very promising anti-cancer compound Minnelide. This small molecule is a synthetic prodrug of Triptolide, a diterpenoid epoxide endogenously produced by the thunder god vine, *tripterygium wilfordii*, which binds XPB and inhibits its ATPase activity [7]. Minnelide is currently in Phase I clinical trials and shows activity against several gastrointestinal cancers [2]. Several proteins were reported to bind to Triptolide but only mutations in the Cys342 of XPB conferred to cancer cells their resistance to this drug [4], validating XPB as its main target *in vivo*. Through the inhibition of the ATPase activity of XPB, Triptolide and Minnelide probably inhibit the translocation of XPB downstream of the transcription start site thereby disrupting the release of the transcriptional block imposed by XPB itself. Similar observations concerning the involvement of XPB in basal transcription have been made in the last months [5, 6]. They confirmed that basal transcription can take place without XPB but some of these data also indicated that the absence of XPB impacts specific transcription programs. For instance, it was shown that expression of genes that were targeted by the viral protein “tat” depended on the presence of XPB [6]. It is not known why XPB would become needed when “tat” protein is involved but this model is of interest to the further understanding of the different functions of XPB in transcription initiation.
